# Micromachining on and of Transparent Polymers for Patterning Electrodes and Growing Electrically Active Cells for Biosensor Applications

**DOI:** 10.3390/mi8080250

**Published:** 2017-08-15

**Authors:** Chandana Karnati, Ricardo Aguilar, Colin Arrowood, James Ross, Swaminathan Rajaraman

**Affiliations:** 1 Alcon Laboratories, Duluth, GA 30097, USA; chandana.karnati@gmail.com; 2 Intel Corporation, Hillsboro, OR 97124, USA; rjaguilar7@gmail.com; 3 Axion BioSystems Inc., Atlanta, GA 30309, USA; carrowood@axion-biosystems.com (C.A.); jross@axion-biosystems.com (J.R.); 4 NanoScience Technology Center and Bridging the Innovation Development Gap (BRIDG), Departments of Material Science and Engineering and Electrical and Computer Engineering, University of Central Florida, Orlando, FL 32826, USA

**Keywords:** polymer micro/nanofabrication, biosensor arrays, microelectrode arrays, assembly/packaging processes, biofunctional devices

## Abstract

We report on microfabrication and assembly process development on transparent, biocompatible polymers for patterning electrodes and growing electrically active cells for *in vitro* cell-based biosensor applications. Such biosensors are typically fabricated on silicon or glass wafers with traditional microelectronic processes that can be cost-prohibitive without imparting necessary biological traits on the devices, such as transparency and compatibility for the measurement of electrical activity of electrogenic cells and other biological functions. We have developed and optimized several methods that utilize traditional micromachining and non-traditional approaches such as printed circuit board (PCB) processing for fabrication of electrodes and growing cells on the transparent polymers polyethylene naphthalate (PEN) and polyethylene terephthalate (PET). PEN-based biosensors are fabricated utilizing lithography, metal lift-off, electroplating, wire bonding, inkjet printing, conformal polymer deposition and laser micromachining, while PET-based biosensors are fabricated utilizing post-processing technologies on modified PCBs. The PEN-based biosensors demonstrate 85–100% yield of microelectrodes, and 1-kHz impedance of 59.6 kOhms in a manner comparable to other traditional approaches, with excellent biofunctionality established with an ATP assay. Additional process characterization of the microelectrodes depicts expected metal integrity and trace widths and thicknesses. PET-based biosensors are optimized for a membrane bow of 6.9 to 15.75 µm and 92% electrode yield on a large area. Additional qualitative optical assay for biomaterial recognition with transmitted light microscopy and growth of rat cortical cells for 7 days *in vitro* (DIV) targeted at biological functionalities such as electrophysiology measurements are demonstrated in this paper.

## 1. Introduction

Cell-based biosensors and biofunctional devices such as microelectrode arrays (MEAs, also called multielectrode arrays or micromachined probes) have become invaluable tools for scientific discovery, biological multifunctional characterization and medical advancement. Since they can actively manipulate and monitor cellular activity both at the single cell and cellular network levels, they provide extraordinary insight into complex neural interactions [1]. Today first-generation biosensor arrays are used in applications as far ranging as drug screening, toxicity testing, cardiac pacing and epilepsy research [2,3,4,5]. Due to the size of cells involved, which are in the scale of microns, micro/nanofabrication is an attractive technique for developing and manufacturing such biofunctional sensors. Several microfabricated biosensor MEAs have been reported since 1969 for multifunctional characterization such as extracellular stimulation and recording from patterned electrically active cells (such as neuronal cells, stem cells, cardiac cells etc.) on these sensor arrays [6,7,8].

Silicon has served as the substrate of choice for the microfabrication and assembly of such biosensor arrays (applied mainly toward *in vivo* neuroscience, and *in vivo* pharmacology though silicon-based *in vitro* MEAs have also been reported) due to mature microfabrication technologies, the ability to integrate complementary metal oxide semiconductor (CMOS) electronics technology with MEA technology, and advanced tool sets that exist for the fabrication of MEAs manufactured from silicon [9,10,11,12,13]. Two-dimensional, three-dimensional, and CMOS-integrated biosensor arrays have been reported by several groups. Successful commercialization of such devices has been reported as well [14,15]. However, when applying MEA technology to *in vitro* biological assays in neuroscience, cardiology, pharmacology etc., transparency for inverted microscopy becomes an important biofunctionality consideration since traditional *in vitro* cell cultures are observed, monitored and manipulated from the bottom-side [16]. Transparency additionally enables *content-based assays* (optical images) in addition to *information-based assays* (electrical signals), opening up new multifunctional applications for these devices. As a result, there is a need for *transparency* in the substrate of choice utilized to fabricate MEAs. Since silicon is not transparent, over the last 35 years several groups have reported the microfabrication of MEAs out of glass [7,17,18]. Thomas et al. report the microfabrication of two-dimensional (2D) MEAs on glass cover slips by depositing and defining layers of metal and insulation respectively utilizing surface micromachining processes [7]. In fact, this paper is the first report of *transparent MEA* utilized for neuroscience. Several other groups have since reported similar technologies for the microfabrication of MEAs [19,20,21]. Heuschkel et al. report the fabrication of three-dimensional (3D) glass MEAs by undercutting a chromium mask with wet etching of glass to create 3D pyramids (less than 100 µm tall) on which metal and SU-8 insulation layers are subsequently defined [18].

**Figure 1:**
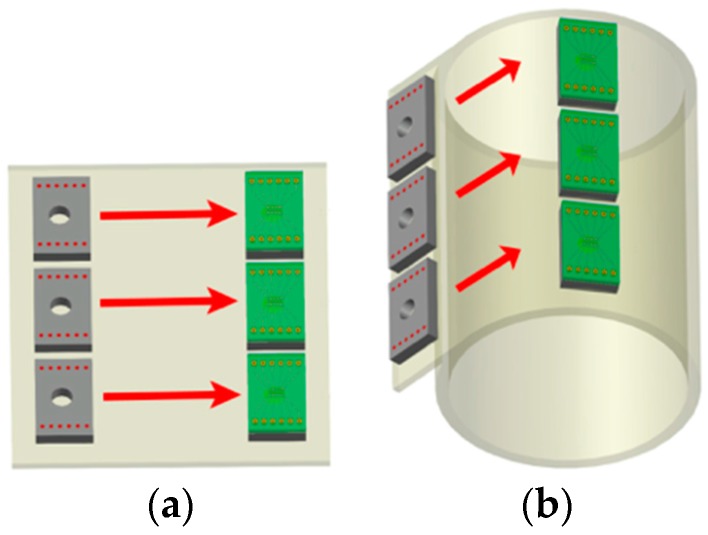
Schematic of reel to reel (**b**—right hand side) and panel-based (**a**—left hand side) fabrication approaches. The schematic indicates that as the polymer goes through a step, multiple biofunctionalities are printed on the polymer for sensing applicatiosns.

As the application areas for such cell-based biofunctional sensors increase, the disposable nature of the device becomes increasing important. Even though glass-based microfabrication is an effective, transparent technology for the development and manufacturing of biosensor microelectrode arrays, disposability is hard to achieve and processes such as “through glass vias” are still in their infancy. Cost-effective technologies that can either minimize the usage of cleanroom-based processes and substrates or perform processing in large-area formats for low-cost micromanufacturing are key toward such a pursuit. Printed circuit board (PCB) processing is a likely candidate for such approaches since it satisfies both criteria using large area processes that are performed outside the cleanroom. Giovangrandi et al. [22], report an unmodified printed circuit board (PCB) process that has been utilized to fabricate *low-cost* biosensors for disposable neural and cardiac biosensing applications. They utilize all standard PCB process steps such as drilling, photo printing, via plating, electroless plating and laser micromachining to fabricate relatively large (~75–100 µm electrode size) microelectrodes. Such sensors take advantage of panel-based fabrication approaches (the typical size of the panel being 18 inches by 24 inches), dramatically reducing costs when compared to wafer-based processes. Since this process is an unmodified PCB approach, FR-4 and Kapton are used as substrates for the MEA. It is an efficacious technique for micromanufacturing MEAs and excellent results with cardiac cultures are reported in this paper, but there are some down sides to this approach. The substrate material, FR-4 or Kapton, are neither transparent nor biocompatible [23]. Kapton has been shown to be biocompatible with respect to certain cell lines [24] but also fragile for long-term applications [25]. The problem of transparency still exists even though there have been efforts to develop transparent polyimides in recent years [26,27]. Thus, there exists an extensive but unmet demand for cost-effective cell-based biofunctional sensor arrays such as MEAs that can be produced from alternative microfabrication approaches that utilize technologies from the PCBs and packaging disciplines along with Micro Electro Mechanical Systems (MEMS) processes. Additionally, if these approaches take advantage of reel-to-reel or roll-to-roll processing or panel-based approaches (Figure 1), they can truly produce cost-effective, transparent biosensor arrays that can be “*used and tossed”* or made *entirely disposable*.

**Figure 2:**
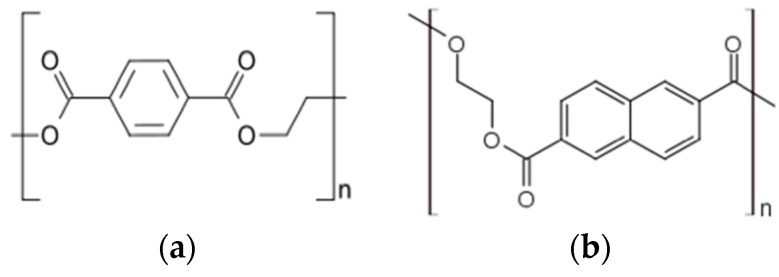
Chemical structure of polyethylene terephthalate (PET) (**a**) and polyethylene naphthalate (PEN) (**b**). Adapted from DuPont Teijin datasheets.

We report micromachining and assembly/packaging development for two such approaches with transparent polymers, polyethylene terephthalate (PET) and polyethylene naphthalate (PEN). Both of these polymers exhibit excellent transparency [28] in the visible spectrum while demonstrating superior biocompatibility [29] and hence are suitable candidates for microfabrication of biofunctional sensors. Their chemical structures exhibit remarkable stability (Figure 2), a property that has resulted in polyester-based materials being increasingly used in food and water packaging [28]. PET is introduced as a biocompatible, transparent polymer utilized for such an application for the first time (to our knowledge). PET has been utilized in plastic MEMS, chemiresistors, supercapacitors, and implantable bioelectronic interfaces [30,31,32,33] but not *in vitro* biosensor arrays such as MEAs. Similarly, PEN is a common substrate that has been utilized for applications such as flexible displays [34,35], energy storage capacitors [36], organic light-emitting diodes [37], solar cells [38] and other flexible electronics applications [39] but to date not for the fabrication of biosensor MEAs. Additionally, due to its two condensed aromatic rings, PEN is typically considered for applications which require improved strength, thermal stability, elastic modulus, and chemical and hydrolytic resistance when compared to PET [40]. We report micromachining processes for PCB/MEMS-based fabrication and assembly approaches to demonstrate both PET-based and PEN-based two-dimensional biofunctional sensor arrays. The PET-based MEAs are introduced in a high-throughput (HT) format in an American National Standards Institute (ANSI)/Society for Lab Automation and Screening (SLAS)-compliant substrate [41]. Such HT biosensor interfaces have been shown to reduce the time for experimentation for toxicity studies by an order of magnitude or more compared to existing low-throughput interfaces [2]. The PEN-based MEAs that are introduced in a traditional single-well format though scaling to a HT format based on the processes developed in this paper should be feasible.

The Materials and Methods section describes the design considerations for both the PET and PEN-based approaches. Additionally, the microfabrication and assembly technologies developed for patterning electrodes on both PEN and PET are detailed. The characterization methods are also described in this section. The Results and Discussion section summarizes the results obtained from the various characterization of the processes and materials involved in the construction of biosensor arrays such as MEAs. We finally conclude the paper with a summary of our findings.

## 2. Materials and Methods

In this section, the design, microfabrication and assembly materials and methods developed for patterning electrodes on both PEN and PET are discussed in detail.

### 2.1. Design of PEN-Based Biosensor Arrays

The PEN-based biosensor MEAs were designed to fit an Axion BioSystems’ single-well Muse^TM^ commercial system [20]. The overall size of the MEA is designed to be 19.4 mm by 19.4 mm. The MEA is designed to contain a single well of 64 microelectrodes in an 8 × 8 grid format with integrated ground (or reference) electrodes for both stimulation and recording purposes. The microelectrodes are 30 µm in diameter and have a pitch of 200 µm (in both *X* and *Y* directions) separating two electrodes. The integrated ground or reference electrodes (as opposed to external ground electrodes) are designed to be approximately 40 times the size of the microelectrodes. The microelectrodes are confined in an area of roughly 1.45 mm × 1.45 mm in the center of the well. The microelectrodes are connected to the “external world” using through-plated vias on a printed circuit board (PCB), which serve as the connection mechanisms between the microelectrodes and Axion’s Muse electronics.

### 2.2. Design of PET-Based Biosensor Arrays

The PET-based biosensor MEAs were designed to fit American National Standards Institute (ANSI)/Society for Lab Automation and Screening (SLAS) specifications [41]. These design constraints were introduced in order to enable these sensor arrays to be compliant with high-throughput instrumentation such as plate readers, robotic liquid handlers etc. By accomplishing such compliance, the ease of integration of these MEAs into other high-throughput instrumentation becomes seamless. The overall size of this MEA was designed to be 3.21 inches by 4.86 inches (81.534 mm × 101.6 mm) and it was structured to accommodate 12 wells with microelectrodes. Each well contains 64 microelectrodes in an 8 × 8 grid format and four integrated ground (or reference) electrodes. The microelectrodes are 30 µm in diameter and have a pitch of 200 µm (in both *X* and *Y* directions) separating the two electrodes. The ground electrodes are designed to be approximately 40 times the size of the microelectrodes. In excess of 780 plated through vias obtained utilizing PCB approaches are designed to serve as the “connection mechanisms” between the microelectrodes/grounds and the electronics/software (Axion’s Maestro^TM^ electronics not described in this work) both on the bottom and top sides of the substrate.

### 2.3. Microfabrication and Assembly Technologies for PEN-Based Biosensor Arrays

The PEN-based MEAs are proposed to be developed on a reel-to-reel or roll-to-roll fabrication format. Even though we have developed all the processes on a 4-inch by 4-inch substrate, we believe with minimal modifications to the processes developed, this technology can be made compatible with large-area, cost-effective micromanufacturing techniques (not demonstrated in this work).

#### 2.3.1. Printed Circuit Board (PCB) Design and Fabrication

**Figure 3:**
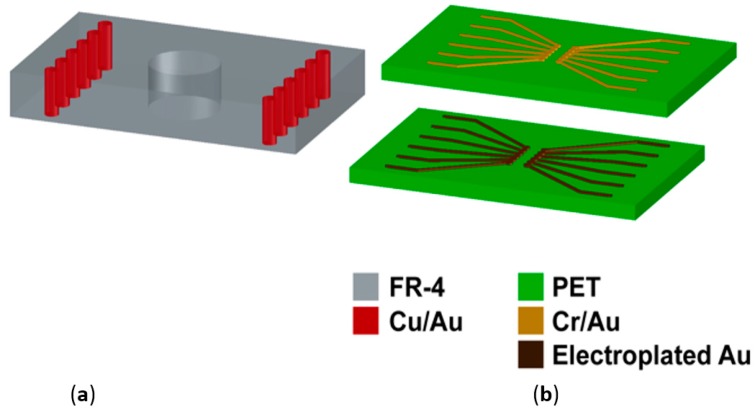
Schematic of the fabrication of the printed circuit board (PCB) utilizing standard processes (**a**) and metallization on PEN (**b**). The PCB is fabricated with plated through metal vias for top to bottom interconnects. The PEN film is processed with thin film metallization and electroplating.

The printed circuit board (PCB) was designed using CADSoft Eagle (*CADSoft*, Pembrooke Pines, FL, USA) with the following process specifications: 5 mils (127 µm) minimum metal trace width; 5 mils (127 µm) minimum spacing between traces; and 12 mils (304.8 µm) minimum plated through via dimension. For fabrication, standard flex-rigid PCB processes with metal layers on both sides of the FR-4 epoxy composite were utilized [42]. The steps used in the process are all standard (e.g., electroless plating, photodefinition, etching, routing, via drilling and lamination) and hence the PCBs were fabricated at a commercial vendor (Innovative Circuits, Alpharetta, GA, USA). An electroless nickel/immersion gold (ENIG) finish (~5 µm nickel and ~0.2 µm gold) was used as the top coating on the metal pads on the PCB. Figure 3a is a schematic of the side view of a single layer PCB with interconnected metal from the top to the bottom sides.

#### 2.3.2. Definition of Microelectrodes on PEN Substrates

Figure 3b depicts the process flow for the definition of metal on PEN. The first step in the fabrication of PEN-based biosensor MEAs is the definition of metal traces on PEN. We utilized well-characterized lift-off techniques to achieve thin film definition on PEN. As a first step in the process, a 4-inch by 4-inch sheet of 5 mil (127 µm)-thick PEN was attached on a glass substrate utilizing Kapton tape. A layer of ~6-µm photoresist (NR9-3000PY, Futurrex Inc., Franklin, NJ, USA) was spin-coated and soft baked for 150 °C for 2 min in an oven. The photoresist was exposed with the metal trace pattern using a UV light source (Karl Suss MA-6; energy used, 1150 mJ) and developed using RD6 (Futurrex Inc., development time, 35–40 s) after a post-exposure bake for 2 min at 100 °C. A short plasma treatment (Plasma Therm RIE: CHF_3_ 5 sccm, O_2_ 45 sccm, power 200 W, pressure 200 mTorr, time 30 s) preceded metal deposition on an E-Beam evaporator (CHA Industries, Fremont, CA, USA). A metal stack (50 nm of Ti and 500 nm of Au) was deposited on the PEN substrate. Metal trace definition was achieved using an overnight passive (just soaking) lift-off process in acetone.

**Figure 4:**
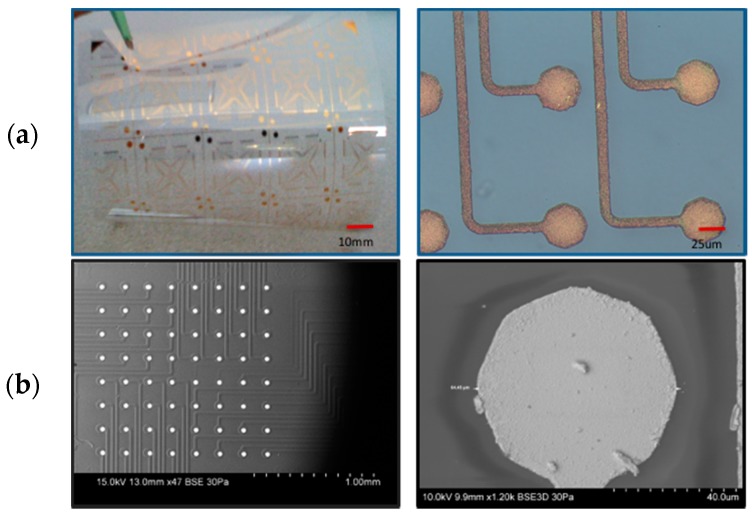
Optical (**a**) and variable pressure SEM (**b**) micrographs from definition of electrodes on PEN. Images from 4a depict a flexible PEN sheet of MEA “die” and a close up optical image of electroplated gold electrodes prior to insulation definition. Images from 4b depict SEM images of the insulation defined gold electrodes with an array on the left and a single electrode on the right.

The second step in the process was electroplating a layer of gold on the defined thin film gold traces. The metal traces as defined by lift-off are terminated in a large rectangular pad (size 500 µm), which is intended to be used as an electroplating anchor pad to be disconnected post electroplating. Gold is electroplated to serve as microelectrode material on the entire chip (all metal traces are electroplated in this step). Techni-Gold 25 ES (Technic Inc., Cranston, RI, USA) is used as the ready-to-use neutral, non-cyanide electroplating solution for this particular step. The solution is heated up to 65 °C and a neutral pH of 7–7.2 is maintained throughout the process. A current of −10 mA is applied across the entire chip and a counter electrode (platinum wire) is utilized during the electroplating process. Even though different plating times were evaluated, the best results were obtained at 5 min of electroplating. These parameters are targeted at achieving approximately 5-µm-thick electroplated gold material (according to the manufacturer’s datasheet). The rectangular anchor pad is disconnected and the metal traces/electrodes defining an individual chip are singulated using a CO_2_ laser (Hermes Gravograph, Duluth, GA, USA) micromachining process. Insulation definition is achieved after the packaging of PEN substrates described in Section 2.3.5. Figure 4 is a collage of optical and SEM micrographs of the various processes developed on PEN substrates detailed in this section.

#### 2.3.3. Inkjet-Printed Vias for Assembly of PEN Biosensors

**Figure 5:**
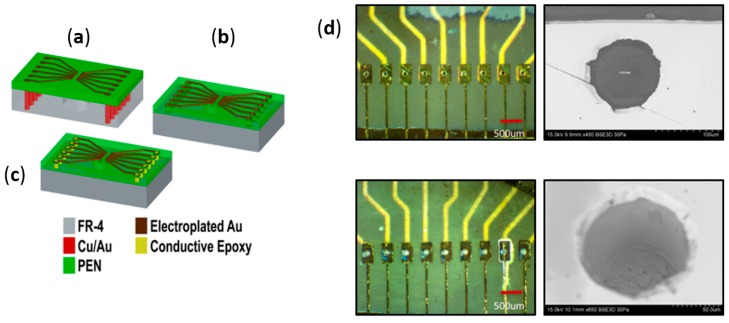
Inkjet printing process flow: (**a**) attach PEN biosensor “die” to the PCB; (**b**) UV laser micromachining of PEN to create vias; (**c**) inkjet printing of vias. Optical and SEM micrographs of the fabricated structures (**d**) depicting the vias before on the top and after inkjet printing on the bottom.

The PCB developed in Section 2.3.1 and the individualized PEN substrates realized in Section 2.3.2 are combined utilizing a die attach process wherein the microfabricated PEN substrate is attached to the PCB utilizing two-part epoxy (Masterbond, Hackensack, NJ, USA). In order to establish connections between the gold pads on the PEN substrate and the gold pads on the PCB substrate, two separate technologies were developed. The first technology involves the usage of inkjet printing of conductive ink to define the vias. The second technology utilized wirebonding and it is described in Section 2.3.4. Figure 5a–c depicts the schematic for inkjet-printed packaging process for PEN MEAs. The first step is the definition of vias in the PEN substrate. Via definition was accomplished utilizing UV laser micromachining. An excimer laser (Resonetics Inc., Nashua, NH) operating at 248 nm was used for this particular step. It is an excellent tool for such a process as it can be utilized to selectively ablate PEN stopping on thick metal as described in some of our previous work [43,44]. A laser pulse of 200 mJ at a frequency of 90 Hz was used in this process. Additionally, 1000 bursts at a 50% transmission were used to ablate through the PEN material. Once the vias were ablated, we utilized the Microfab JetLabII Inkjet printer (Microfab Technologies, Plano, TX, USA) which is a table-top inkjet microdispensing unit ideal for non-contact printing of several materials including conductive epoxies. UT Dots’ UTDAg conductive silver nanoink (UT Dots Inc., Champaign, IL, USA) was utilized in this printing process. We printed 50 dots/via of the UTDAg nanoink at a frequency of 600 Hz to achieve conductivity through the thickness of the PEN substrate (~125 µm thick). Figure 5d depicts optical and SEM micrographs of the printed vias. We were able to achieve ~85% conductivity of the interconnect for every device developed in this process using this recipe. Even though inkjet printing was a successful process with a demonstrated high yield, the throughput of the process was rather slow due to the need to repeatedly clean the micronozzle used in the printing. As a result, we started evaluating alternate approaches to achieve electrical connections from the metal on PEN to metal on the PCB.

#### 2.3.4. Wire Bonded Interconnects for the Assembly of PEN Biosensors

**Figure 6:**
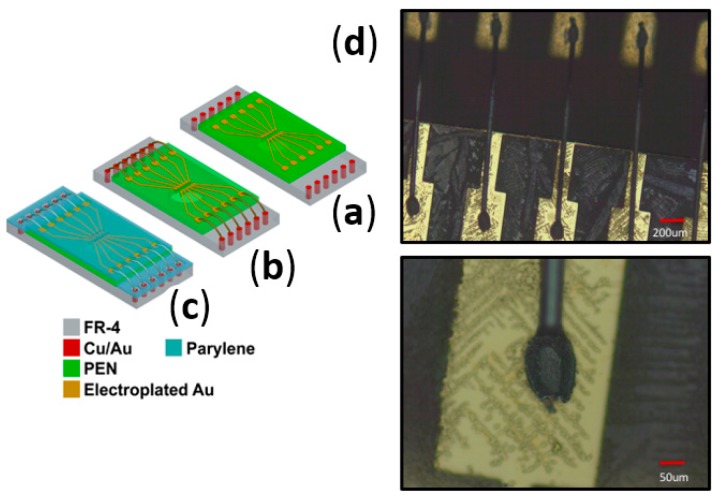
Wire bonding process flow (**a**–**c**) and optical micrographs of wire bonds on a PEN device (**d**).

Figure 6a–c depicts the schematic for a wire bonding process to interconnect bond pads on the PEN surface (electroplated Au on thin film Ti/Au) with bond pads on the PCB surface (Electroless Nickel Immersion Gold—ENIG finish on electroplated copper). Wire bonding has been utilized as a successful technology in several chip-on-board packaged devices including MEA biosensors [45] and as a result, it was evaluated as a possible interconnect technology for PEN biosensor MEAs. The Kulicke and Soffa Wedge Bonder K&S 1472 (Kulicke and Soffa Industries, Singapore) was utilized for wire bonding. It is a wedge bonder configured with 1-mil (25.4 µm)-thick aluminum wire and can be operated in a fully automatic mode after training the tool for a particular wire bonding process. As a result, the 72 interconnections on the device can be wire-bonded in a matter of less than a min. The power utilized in the wire bonding process ranged from 90–110 mW and the dwell time for the bond varied from 45–60 ms. We were able to achieve 100% connectivity of the interconnect with the optimized process above. Figure 6d depicts optical micrographs of the wire-bonded PEN MEAs.

#### 2.3.5. Definition of Insulation for PEN Biosensors

Figure 6a–c depicts the definition of insulation on PEN biosensor MEAs. Insulation is the final step in the process of the microfabrication of PEN MEAs. Parylene has been selected as the conformally deposited insulation material in this process. Commercial 2-D glass MEAs typically have SU-8 or silicon nitride as insulation materials [19,20]. SU-8 can most certainly be substituted for Parylene in PEN MEAs but we chose to simplify PEN chip fabrication and utilize Parylene as the insulation material of choice. Parylene has added advantages of being biocompatible and ease of processing (conformal deposition at room temperature which is compatible with polymer processing). Additionally, parylene can be deposited conformally after the assembly processing has been completed encapsulating the biosensors and the packaging, thus providing fabrication flexibility. We deposited 5 µm on both types of assembled biosensor arrays utilizing an SCS Parylene Deposition System (Specialty Coating Systems, Indianapolis, IN, USA). An excimer laser (Resonetics Inc., Nashua, NH, USA) operating at 248 nm was used to define the microelectrodes after parylene deposition. Such selective ablation of parylene on thick metal for the definition of microelectrodes (30 µm in diameter) has been demonstrated previously by us [43,44]. Figure 4b depicts an SEM image of all 64 microelectrodes clearly visible in a variable pressure SEM (Hitachi S-3700N, Hitachi High-Technologies, Krefeld, Germany) on a PEN-based biosensor array.

### 2.4. Microfabrication and Assembly Technologies for PET-Based Biosensor Arrays

The PET-based biosensor MEAs are proposed to be developed on a panel-based fabrication format. The assembly process development work including the PCB fabrication was performed on a 12 inch × 18 inch panel while the microfabrication process development was performed on an ANSI/SLAS substrate size of 3.21 inches by 4.86 inches (81.5 mm × 101.6 mm).

#### 2.4.1. Printed Circuit Board Design and Fabrication

**Figure 7:**
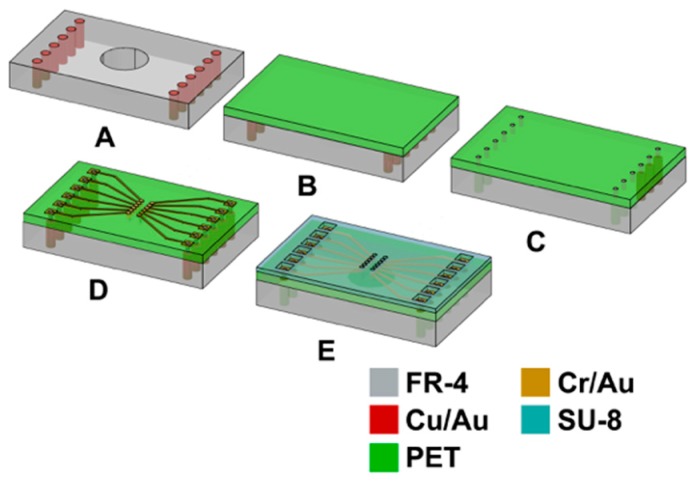
Fabrication process flow schematic for PET-based biosensor arrays. The process flow depicts the integration of PET on PCBs (Steps **A**–**C**) and post processing the PET layer (steps **D** and **E**). The process flow schematic depicts only one 5-mm through hole but there are a total of 12 of these holes in a single biosensor MEA.

The printed circuit board (PCB) was designed using CADSoft Eagle (*CADSoft*, Pembrooke Pines, FL, USA) with the following process specifications: 5 mil (127 µm) minimum metal trace width; 5 mil (127 µm) minimum spacing between traces; 12 mil (304.8 µm) minimum plated through via dimension. For fabrication, standard flex-rigid PCB processes with metal layers on both sides of the FR-4 epoxy composite were utilized [42]. Figure 7 depicts the schematic of the side view of the process flow for construction of PET-based biosensor arrays including both the microfabrication and assembly steps. The sequences used in the process are all standard with the exception of utilizing a top flex layer of 3 mils (76.4 µm) of transparent, biocompatible polyethylene terephthalate (PET; trade name Mylar, *DuPont-Teijin*, Hopewell, VA, USA) which is non-standard in a flex-rigid PCB environment. Kapton^®^ is the standard layer that acts as the flex component of a flex-PCB or a flex-rigid process but the required transparency for inverted microscopy necessitates the use of PET in our process. Additionally, to accommodate the need for inverted microscopy, 12 through-holes were drilled in the rigid part of the PCB (5 mm in diameter) at the microelectrodes’ location with the PET membrane suspended on top of the opening in the FR-4 substrate.

**Figure 8:**
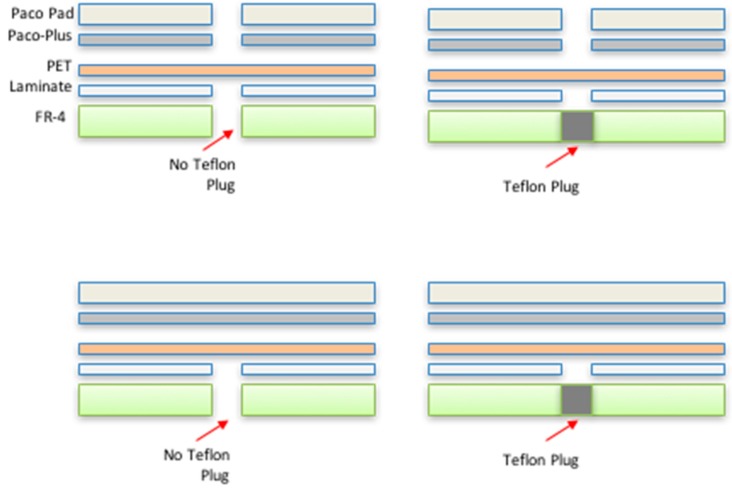
Schematic of the lamination process development. In addition to the functional layers (PCB, laminate or adhesive and PET), non-functional additions (pads and Teflon) are used in a variety of combinations depicted above.

For bonding of the PET membrane to the PCB, the PET film was first treated to a barrel oxygen plasma cycle for 5 min. This short treatment temporarily activated the surface of the PET and assisted in better lamination with the standard flex-rigid adhesive (1 mil acrylic; trade name Pyralux LF 0025, *DuPont,* Wilmington, DE, USA). The lamination was carried out in a heat press at 120 °C at a pressure of 300–350 psi. The total process time (including heating up and cooling down of the lamination press) was around 4 h for a panel (size 12 inches × 18 inches) that can accommodate multiple PET MEAs. Various techniques were used as follows: (a) Openings in supporting pads for lamination; (b) Presence and absence of a Teflon plug used to “planarize” PET in the 5 mm opening during lamination; and (c) No openings in support pads for lamination. These techniques were evaluated for minimizing the PET membrane bow that typically develops in such a process. Figure 8 illustrates the schematics of the techniques described above. The technique selected (based on bow data results described in Section 3.2.1) involved the creation of openings in the support pads in proximity to the microelectrode areas during lamination (bow data in Section 3.2.1). As the final step of the flex-rigid PCB fabrication, laser vias were drilled using a UV laser (248 nm) in the PET/adhesive layer and stopping on underlying metal layer [43,44]. These laser vias (~200 µm in diameter) serve as the access ports for the interconnection of flex-rigid PCB vias and microfabrication metal traces (described in the next section). A metal layer of electroless nickel/immersion gold (~5 µm nickel and ~0.2 µm gold) was deposited on the traces and the vias to complete the fabrication of the flex-rigid PCBs. This layer is typically deposited to prevent copper migration but in our case it helps with better adhesion of microfabrication metal traces (described in the next section) to the PCB traces.

#### 2.4.2. Post Processing of PET PCB for Definition of Microelectrodes

The first step in post processing of the flex-rigid PCBs is removal of laser micromachining residue from the vias micromachined with the UV laser. Techniques such as Reactive Ion Etching (RIE), passive oxygen plasma, solvent cleaning and combinations of these processes were tested for residue removal. Results of the efficacy of the techniques evaluated are detailed in Section 3.2.2. A combination of solvent sonication (Branson 5510 Sonicator; Room Temperature) followed by RIE (Plasma Therm RIE; CHF_3_: 5 sccm; O_2_: 45 sccm; power: 150 W; pressure: 150 mTorr) was chosen as the best technique for removal of residue with minimal damage to the optical transparency properties (transparency measured qualitatively with an optical assay as described in Section 3.2.5) of the PET membrane.

**Figure 9:**
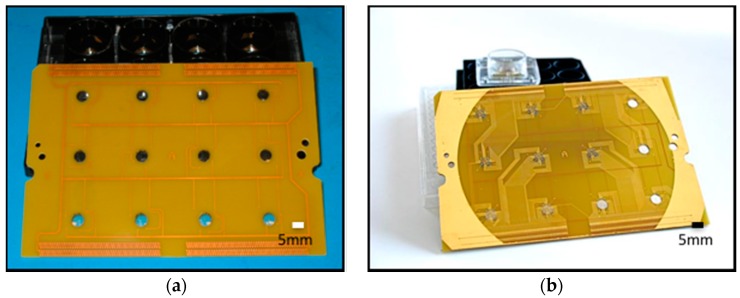
Optical micrographs of PET-based Printed Circuit Board (PCB) (**a**) and a fully post-processed biosensor array (**b**).

Microfabrication process on the flex-rigid PCB is summarized in Figure 7D,E in a side view process flow representation. The first step is the definition of metal traces, which serve as leads for the microelectrodes. The metal traces have to be defined continuously through the depth of the laser micromachined vias (~100 µm deep; 75 µm PET plus 25 µm acrylic) and on the PET membrane itself. Various techniques were evaluated (with impedance yield and minimal damage to PET transparency as process objectives) and the efficacy of these techniques is summarized in Section 3.2.2. A PVD filament evaporator with substrate rotation (*Kurt J. Lesker Company*, Clairton, PA, USA) deposition of 30 nm of chromium followed by 350 nm of gold was found to be the most efficient method for metal deposition on the PET substrate that met our goals. A short plasma treatment (Plasma Therm RIE; CHF_3_: 5 sccm; O_2_: 45 sccm; power: 200 W; pressure: 200 mTorr; time: 30 s) preceded metal deposition and served to activate the PET surface for better adherence of metal on PET. The deposited metal was defined by spin coating a layer of positive resist (Shipley SC 1827; thickness of 3 µm; *Shipley Company Inc.*, Marlborough, MA, USA) and UV lithography (Karl Suss MA-6; energy used: 400 mJ; development time: 1 min in an MF 319 developer). The metal stack was etched using GE 8148 gold etchant (*Transene Company*, Danvers, MA, USA) and chrome etchant (CR-7S, *Transene Company*, Danvers, MA, USA). The average etch times were optimized for minimizing the undercut and metal removal in vias. These etch times were 2 min for the gold and around 1 min for the chromium layers. Microelectrodes were defined on the metal traces by spin coating a layer of 5 µm SU-8 and then defining it using standard UV lithography (Karl Suss MA-6; Energy used: 49 mJ/cm^2^; development time: 20–25 s in Thinner P developer supplied by *MicroChem*, Newton, MA, USA). An oxygen plasma treatment (Yes R1 Plasma Cleaner; O_2_ flow rate 1.5–2.0 CFM; time: 1 min) preceded the SU-8 coating step. It was found that this process improved the adhesion of the SU-8 material to the PET surface and prevented the SU-8 from delaminating from the bulk PET surface. Figure 9a,b depict optical micrographs of a fabricated PCB board with the PET membrane and a completely processed PET flex-rigid sensor array.

#### 2.4.3. Assembly Process Development for PET Biosensors

PDMS culture wells were fabricated by pouring pre-mixed PDMS (Sylgard 184, *Dow Corning*, Midland, MI, USA) in a 10:1 ratio onto 1-inch polystyrene petri dishes with a cylindrical metallic piece placed in the middle in order to mold a donut-shaped culture well structure with a 7-mm inner diameter and a 15-mm outer diameter. The donuts were cured at 56 °C for 5 h before assembly. These PDMS wells were attached to the PET MEA using a thin layer of premixed PDMS and cured for 5 h at 56 °C. This step completed the fabrication and assembly of the PET biosensor arrays.

### 2.5. Characterization Methods for PEN-Based Biosensors

In this section, we outline the methods that were utilized in the characterization of the developed PEN-based biosensor MEAs.

#### 2.5.1. Metal Trace Width Measurement

Metal trace widths of the various designs were measured utilizing a Leica Optical Microscope (Leica DM 2500 M, *Vashaw Scientific*, Norcross, GA, USA) and the Leica Applications Suite software tool. We measured three different metal trace widths in the design that represented the smallest value in the design (15 µm), an intermediate trace width value (40 µm) and a large value (203 µm). Six separate measurements were carried in three different samples to ensure the representation of a wide variety of measurements (*N* = 18 total for each design specification).

#### 2.5.2. Metal Integrity Characterization

The integrity of the metal defined on the PEN substrates were measured after the metal definition steps (both lift-off and electroplating) using a scotch tape peel test (10× repetitions) and observations under the microscope. Additionally, the traces were probed with sharp multimeter tips that served for testing the integrity of the processes as well.

#### 2.5.3. Impedance Characterization of PEN Biosensors

The impedance measurements were performed utilizing the Omicron Labs impedance measurement system (Bode 100, *Omicron Labs*, Klaus, Austria). The Bode 100 tool can serve as a two-channel impedance meter that allows for rapid measurement of the magnitude and phase of microelectrode impedances across a large range of frequencies (0.1 Hz to 100 MHz). The lightweight, portable hardware unit combined with the Bode Analyzer Suite software enables user-friendly impedance measurements rapidly. Impedance measurements are typically performed between the microelectrode, an external reference, or ground electrode (in this case a platinum wire) and cellular conducting media (HBSS, *Invitrogen*, San Diego, CA, USA). The fabricated PEN MEAs were interfaced with the Bode 100 and electrodes were scanned in the frequency range 10 Hz to 100 kHz.

#### 2.5.4. ATP Assay for Cytocompatibility of the PEN Biosensors

The biocompatibility of the PEN-based biosensor was measured utilizing the CellTiter-Glo Luminescent Cell Viability Assay [46]. This assay is a homogeneous method for determining the number of viable cells in culture based on the quantification of the adinosine tri-phosphate (ATP) present. ATP is a well-established indicator of metabolically active cells. The CellTiter-Glo^®^ Assay is designed for use with multiwell formats, making it ideal for automated high-throughput screening (HTS), cell proliferation and cytotoxicity assays. The homogeneous assay procedure involves adding a single reagent (CellTiter-Glo^®^ Reagent) directly to cells cultured in serum-supplemented medium. Cell washing, removal of medium and multiple pipetting steps are not required. The assay system detects as few as 15 cells/well in a 384-well format in 10 min after adding the reagent and mixing.

The homogeneous “add-mix-measure” format results in cell lysis and generation of a luminescent signal proportional to the amount of ATP present. The amount of ATP is directly proportional to the number of cells present in culture. The CellTiter-Glo^®^ Assay generates a “glow-type” luminescent signal, which has a half-life generally greater than five hours, depending on cell type and medium used. The extended half-life eliminates the need to use reagent injectors and provides flexibility for continuous or batch mode processing of multiple plates. The unique homogeneous format avoids errors that may be introduced by other ATP measurement methods that require multiple steps.

For cytotoxicity evaluation one individual PEN biosensor was divided into individual pieces (*N* = 6) such that all the components of the biosensor (parylene, gold and PEN) were represented in each piece. The sensor pieces were added on day one *in vitro* (1 DIV) to a rat cortical neuronal culture (Brain Bits LLC). Serum supplemented media in wells (*N* = 3) was used as a control for the experiment. The ATP assay was performed as described above at 28 DIV and the culture was terminated afterward. The media in the cultures were periodically changed as mandated by the protocol.

### 2.6. Characterization Methods for PET-Based Biosensor Arrays

In this section we outline the methods utilized for the characterization of the PET-based biosensor arrays.

#### 2.6.1. Membrane Bow Measurement of PET-Based Biosensors

The bow on the PET membrane suspended in a 5-mm opening in the FR-4 layer was measured after the flex-rigid PCB fabrication with a P-15 profilometer (*KLA Tencor*, Milpitas, CA, USA) to characterize the various techniques described in Section 2.4.1 (Figure 8) that were evaluated for this purpose.

#### 2.6.2. Via Yield Measurements

Via yields were evaluated across the board (over 780 contact pads) by performing a continuity test on the metalized PET membrane and individual contact pads on the bottom side of the flex-rigid PCB. Metallization yield evaluation was performed using a similar technique as via yields with a slight variation. It was performed after the definition of the metal traces on PET and temporary assembly of the post-processed flex-rigid PCB. Saline (HBSS, *Invitrogen*, San Diego, CA, USA) was used to “connect” all the electrodes in a single well. The electrodes were probed using a multimeter from the bottom side pads to the topside saline.

#### 2.6.3. Metal Integrity Characterization

The integrity of the metal defined on the post-processed PET substrates was measured utilizing a scotch tape peel test (5× repetitions) and observations under the microscope.

#### 2.6.4. Nano-Porous Platinum Electroplating

In order to demonstrate a low-impedance surface coating, a nano-porous layer of platinum was electrodeposited on the microelectrodes using a current of (−4 µA) per electrode in a solution of chloroplatinic acid diluted with HCl and lead acetate (1% chloroplatinic acid; 0.005% lead acetate; 0.01 M HCl, all from *Sigma Aldrich*, St. Louis, MO, USA). A platinum wire (*Sigma Aldrich*, St. Louis, MO, USA) was used as the counter electrode and every electrode was electroplated under DC conditions for 40 s using a current source (Keithley 2400 Source Meter, *Keithley Instruments*, Cleveland, OH, USA).

#### 2.6.5. Optical Assay for Measurement of Transparency

An optical assay was designed to qualitatively study biomaterial recognition on PET substrates after the completion of the fabrication of the biosensor array. We mixed polystyrene microspheres (15 µm in diameter, *Sigma Aldrich*, St. Louis, MO, USA) in PBS solution (*Invitrogen*, San Diego, CA, USA) to an effective concentration of 1 × 10^5^ microspheres/mL. The idea was to develop a rapid assay that can be evaluated in a matter of minutes to simulate cells without culturing procedures which can take up to 24 h before cell attachment can be ascertained. We dispensed such a solution on the post-processed (with metal and SU-8) PET substrates and captured images (in transmitted and fluorescent modes) utilizing an inverted microscope (Nikon Eclipse TE200, *Nikon Instruments*, Melville, NY, USA).

#### 2.6.6. Growing Electrically Active Cells on PET-Based Biosensor Arrays

Primary rat cortical neuronal cells were grown and studied for 7-days *in vitro* on the PET biosensor arrays. The arrays were prepared by a short sterilization cycle that involved cleaning the devices in 90% ethanol followed by a DI water rinse. The sensor arrays were subsequently baked for 5 h at 50 °C in a clean oven. Both the biosensor array and a control polystyrene well plate were coated with 300 µL of 50 µg/mL Poly-d-Lysine for 2 h at 37 °C. Rat cortical neuronal cells (Brain Bits LLC) were immediately plated on the devices at a cell density of 4 × 10^4^ cells/cm^2^. The cell culture media was subsequently added and the cells were maintained in an incubator for 7 days. Media was changed at 4 Days *In vitro* (DIV) and a live/dead assay was performed at 7 DIV.

## 3. Results and Discussion

In this section the characterization results for both the PEN and PET-based biosensor MEAs are detailed and the implications of the results are discussed.

### 3.1. Characterization of PEN-Based Biosensor Arrays

#### 3.1.1. Metal Trace Width Characterization

The results for the metal trace width measurement are as follows: mask design (203 µm) measured 203.01 µm with a Standard Deviation (SD) of 0.3913 µm; mask design (40 µm) measured 42.86 µm with a SD of 0.4555 µm and mask design (15 µm) measured 15.85 µm with a SD of 0.2774 µm. The variation from the design value was found to be the worst for the 40-µm design width (mean: 42.86 µm with a SD of 0.4555 µm) which is a bit perplexing. We believe it could potentially stem from a minor conversion error when the design was converted from the routing design software (CADSoft Eagle) to the mask design software (AutoCAD). The 15 µm and the 203 µm design widths performed much better. Overall, the translation from design to fabrication for thin film metal is well within the acceptable range for such a biosensor application on a new material. Additionally, we measured the thickness of the electroplated gold trace on six different samples (*N* = 3 measurements each for a total of 18 measurements) across the various design widths. We expected a thickness of ~5 µm based on a calculation provided by the manufacturer that involved the deposition rate and time but the mean thickness across 18 measurements was closer to 2.39 µm with a standard deviation of 0.29 µm. The discrepancy could be explained by the fact that the electroplating solution is not well characterized, leading to different experimental and theoretical values. However, the thickness was found to be more than sufficient for the resulting application and the subsequent fabrication steps.

#### 3.1.2. Metal Integrity Characterization

Scotch tape peel tests were performed both after the metal definition on PEN utilizing thin (lift-off evaporation) and thick film (electroplating) techniques. The adhesion of both thin and thick film gold was excellent with no demonstrated delamination for multiple tests (maximum of 10 repetitive tests). Additional probing of the metal traces utilizing a multimeter and sharp probes yielded no change in conductivity (before and after 10× peel tests) demonstrating excellent adhesion of both thin and thick film gold to PEN.

#### 3.1.3. Impedance Characterization of PEN Biosensors

**Figure 10:**
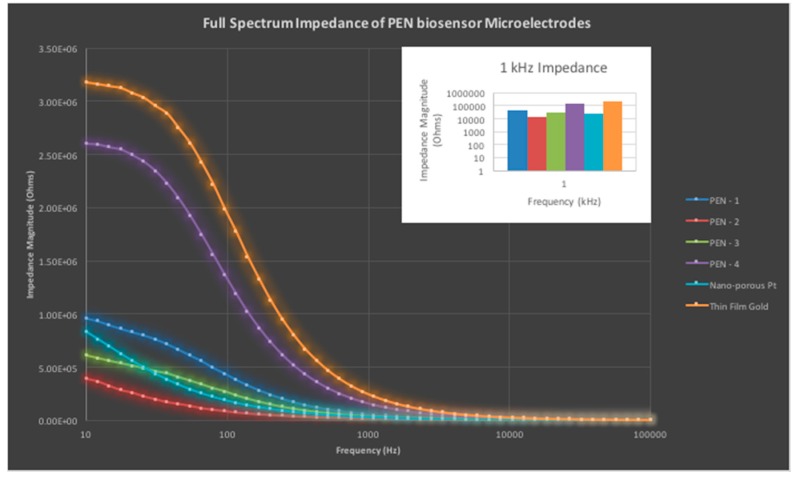
Full spectrum impedance (real part of the complex microelectrode impedance) measurement of PEN biosensors and comparison with nano-porous platinum and thin film gold electrodes. The inset depicts the critical 1-kHz impedance averages for the PEN electrode batches and standard nano-porous platinum and thin film gold electrodes.

Figure 10 depicts characteristic measurements of four groups of electrodes (PEN 1 to 4 in the legend and *N* = 3 for each electrode set) and compares the impedance value to standard, low impedance nano-porous platinum microelectrodes on glass MEAs (*N* = 3, average value represented here) and thin film gold microelectrodes on standard glass MEAs (*N* = 3, average value represented here). The average value of the PEN electrodes is 59.6 kOhms at 1 kHz which is higher than the average value measured for nano-porous platinum electrodes at 26.1 kOhms while being an order of magnitude lower compared to thin film gold electrodes that measured an average value of 183 kOhms at 1 kHz.

#### 3.1.4. ATP Assay for Cytocompatibility of PEN

**Figure 11:**
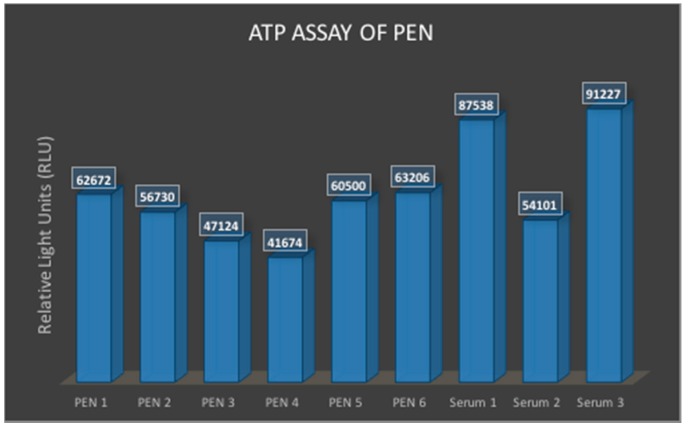
Cytocompatibility measurement of PEN and comparison with standard serum controls utilizing an ATP assay that measures the level of ATP (indicating cell health) using a bioluminescence signal from a plate reader.

The absolute luminescent readings across the *N* = 6 wells from the PEN biosensor material samples and the controls (*N* = 3) are depicted in Figure 11. The figure depicts the following mean and standard deviation values of the bioluminescent signal for the two materials: PEN (mean: 55,317; standard deviation: 8149) and control serum (mean: 77,622; standard deviation: 16,699). We believe that these results represent healthy ATP values for the PEN biosensor MEA materials compared to the serum control though certain experimental difficulties resulted in a large variation in both test pieces. The biofunctionality of the PEN-based biosensor materials is confirmed through this experiment. Longer *in vitro* studies and electrophysiology studies are currently being planned.

### 3.2. Characterization of PET-Based Biosensor Arrays

#### 3.2.1. Membrane Bow Measurement for PET-Based Biosensors

**Figure 12:**
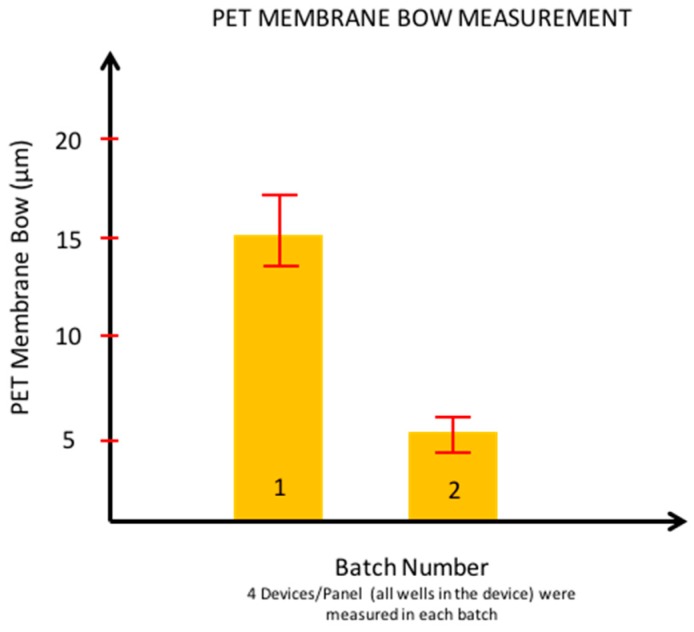
Membrane bow measurement of multiple lots of PET devices. Each lot had *N* = 4 devices and measurements were made across all 12 wells (so an average of 48 wells were measured).

It was found that the bow on the PET membrane with lamination assisted by openings in the padding material to accommodate for the corresponding openings in the FR-4 layer showed the best performance of a concave membrane with minimal bow suitable for post-processing. Figure 12 depicts a graphical representation of the measured bow across two different batches (average of all 12 wells) of flex-rigid PCB boards. This measured bow was found to be lower than 20 µm, which we believe makes the suspended PET membrane suitable for post processing. The other two techniques (with teflon plugs and no holes in padding) created either convex membranes or concave membranes with bowing in excess of 50 µm (data not shown) making these techniques unsuitable for post-processing.

#### 3.2.2. Via Yield Measurements

**Figure 13:**
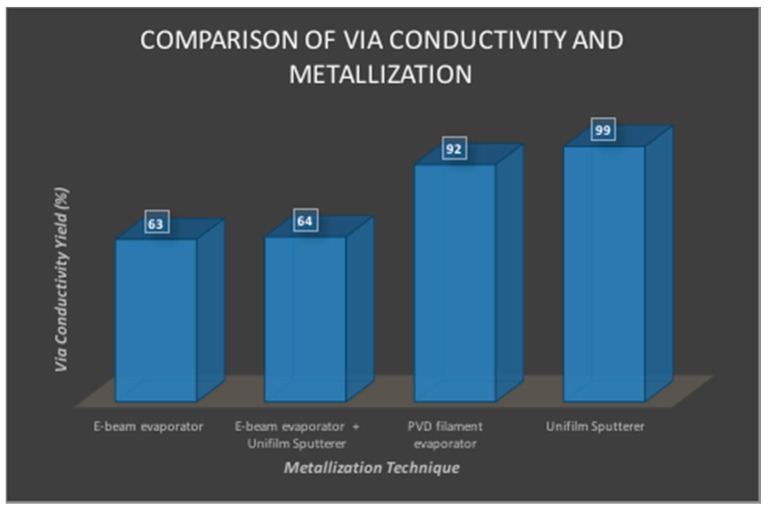
Via yields and metal integrity in PET-based biosensors. These values were calculated for an average of *N* = 3 samples for the various techniques.

Figure 13 depicts via yield measurements after the removal of laser micromachining residue and metallization in the flex-PCB biosensor arrays. For removal of laser micromachining residue, even though good results (85–100% yield) were obtained with almost all the residue removal techniques evaluated, a short solvent (5 min in IPA or methanol) sonication followed by a 15-min RIE was chosen as the best technique in order to minimize damage to the PET membrane. The various metallization techniques resulted in via yields ranging from 63 to 99% and followed expected trends. These trends follow processing physics with e-beam metallization being directional demonstrating the lowest yield of 63% and unifilm sputter metallization and PVD filament resistive heating evaporation with substrate rotation being conformal yielding the best results (>90% yield).

#### 3.2.3. Metal Integrity Characterization

**Figure 14:**
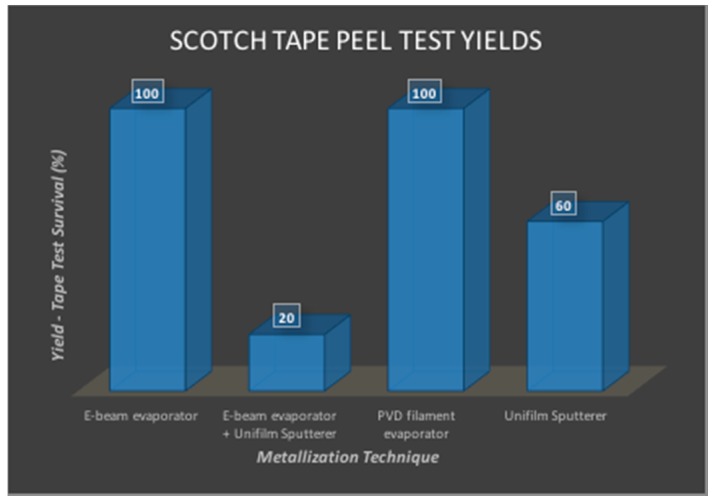
Scotch tape peel test results for the various metallization techniques used. These percentages were calculated for an average of *N* = 5 times for all of the different techniques.

Scotch tape peel tests were additionally performed demonstrating that the metal on the PET film did not delaminate for multiple tests (maximum of 5 repetitive tests; all results normalized to 5× peel tests and expressed as a percentage in Figure 14). PVD filament evaporation showed absolutely no delamination for up to 5× peel testing and hence was chosen as the preferred metallization technique for the PET biosensor MEAs. This technique additionally demonstrates >90% conductivity for the biosensor electrodes across the full substrate (in excess of 780 electrodes per sensor array).

#### 3.2.4. Nano-Porous Platinum Electroplating

**Figure 15:**
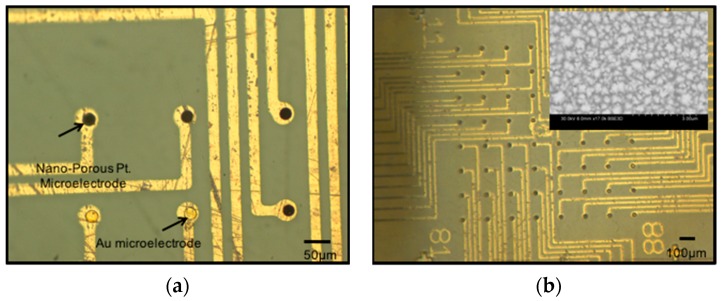
Nano-porous platinum electrodeposited on PET-based biosensor arrays. Optical micrographs depict the comparison between gold and nano-porous platinum (N-P Pt.) electrodes (**a**) and a fully defined N-P Pt. electrode well (**b**) with an inset SEM image depicting the nano-porous structure of the electroplated platinum.

Nano-porous platinum is a suitable non-toxic material for low impedance surface coatings that lowers the impedance of gold microelectrodes to be compatible with neuronal signal acquisition (typically in the 10–100 µV RMS range [11]). This process additionally serves as a technique to evaluate the success of the creation of microelectrodes by combining flex-rigid PCB processes and microfabrication. The electroplating step not only serves to deposit a layer of low-impedance nano-porous platinum on the microelectrodes and ground electrodes but also serves to verify the efficacy of the entire process. The Root Mean Square (RMS) noise level for such coatings typically ranges between 2 and 3 µV (data from our previous work [45]) making these microelectrodes ideal for neuronal signal acquisition. Figure 15 depicts optical and SEM micrographs of nano-porous platinum electroplated on the gold layer in the PET biosensor MEA.

#### 3.2.5. Optical Assay for Measurement of Transparency

**Figure 16:**
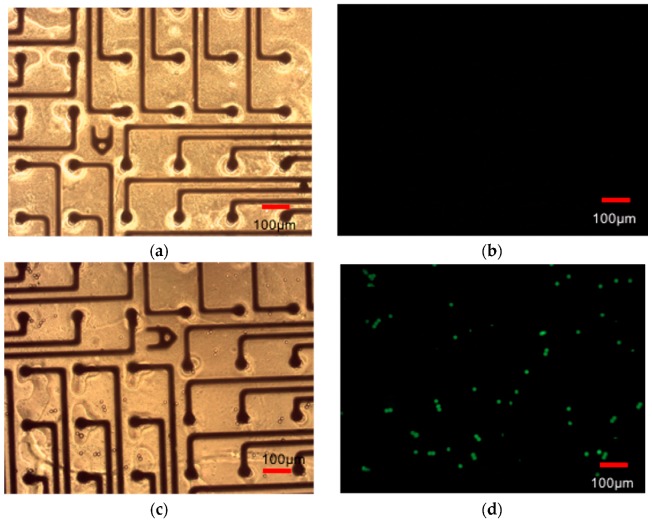
Qualitative optical assay on PET—transmitted brightfield and fluorescent microscopic images before (**a**,**b**) and after (**c**,**d**) addition of polystyrene microspheres.

Figure 16a,b and Figure 16c,d depict images before and after dispensing the polystyrene microspheres on PET biosensor MEAs depicting the ability to visualize these structures on the substrate. This simple proof of concept assay clearly demonstrates promise for visualizing cells on PET biosensor arrays. Quantitative assessment of the concentration of the microspheres was not performed in this basic assay but such an experiment can serve the role of rapidly assessing transparency of polymer materials before and after microfabrication.

#### 3.2.6. Growing Electrically Active Cells on PET-Based Biosensor Arrays

**Figure 17:**
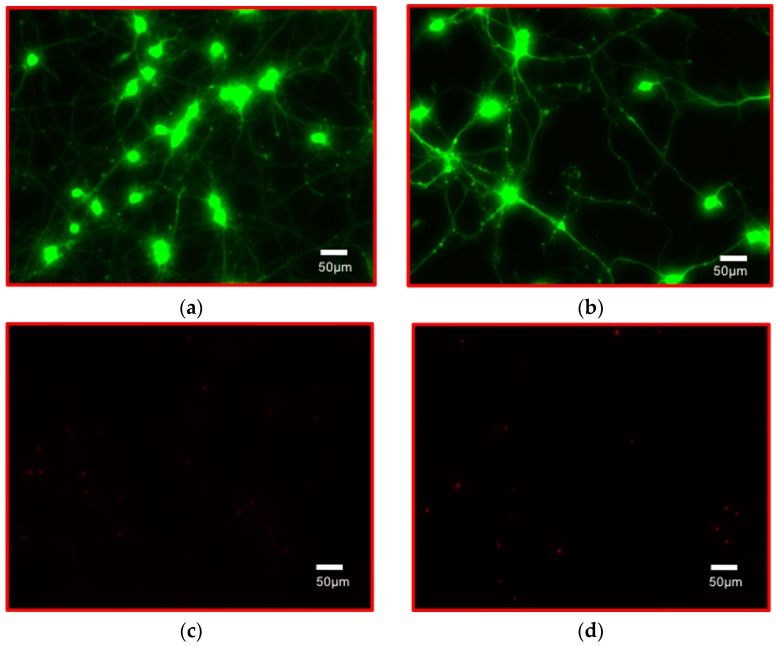
Cell viability assay on PET-based biosensors (7 Days *In vitro* (DIV) florescent images of rat cortical neuronal cells on microelectrode arrays (MEAs) (**b**,**d**) and control PS plates (**a**,**c**). Live staining images (**a**,**b**) and dead staining images (**c**,**d**) depict similar results for both the control and PET MEAs.

Figure 17 depicts sample live and dead staining images from both the control (polystyrene) and the PET biosensor devices depicting excellent neuronal cell definition. Electrophysiological recordings were not performed in this study but they are planned in the near future. The PET biosensor arrays show great promise for electrophysiology and other types of biological measurements toward bioinspired devices and functionalities.

### 3.3. Discussion and Significance of the Results

We have for the first time to our knowledge demonstrated detailed microfabrication and packaging processes for the development of *disposable biosensor arrays* (such as microelectrode arrays or MEAs) on low-cost polymers such as polyethylene naphthalate (PEN) and polyethylene terephthalate (PET). Two separate designs, a single-well design with 64 electrodes and a multiwell design with 768 electrodes have been designed, fabricated, assembled and characterized in this paper. Characterization of the metal trace width and integrity on the PEN-based biosensor arrays depicted that microfabrication steps normally performed on silicon and glass can be adapted to work well on PEN. The impedance of the biosensors defined utilizing electroplated gold was roughly twice the impedance of comparable nanomaterials defined on glass substrates but outperformed other common MEA electrode materials (such as titanium nitride and indium tin oxide) of similar dimensions [19]. The biocompatibility assay results on PEN biosensor arrays depicts promise for future electrophysiological assay development. The PET-based biosensor arrays were a result of a novel co-fabrication approach that combined packaging and microfabrication targeted at overcoming expensive processes when these tasks are performed separately. Processing optimization of such a *co-fabrication approach* have been performed to realize the biosensor MEAs and these efforts resulted in optimized metallization and metal integrity measurements. Traditional nano-porous platinum was electroplated successfully on the MEAs [20,45] and optical transparency for transmitted light microscopy which is a concern for such polymers and growing neuronal cells was established for this new co-fabrication approach that promises to deliver *low cost, disposable but high-throughput MEAs*.

## 4. Conclusions

We have developed multiple microfabrication and assembly processes on biocompatible polymers—polyethylene naphthalate (PEN) and polyethylene terephthalate (PET) toward the integration of cell based biosensors such as microelectrode arrays (MEAs). Both traditional and non-traditional processes such as metal lift-off, electroplating, PCB processes, lamination, inkjet printing, wirebonding, parylene deposition and SU-8 definition have been characterized for the fabrication of such bio-inspired microsensor arrays. We have further characterized these devices by quantifying the results of individual new processes on these materials such as metal integrity, metal trace width and thickness. The full spectrum impedance of PEN-based biosensor arrays was measured and the 1-kHz magnitude of the impedance measured an average of 59.1 kOhms which is comparable to other standard microelectrode materials. The microelectrode yield for PEN-based biosensor arrays was measured between 85 and 100%, making them good candidates for future electrophysiological studies. The cytocompatibility of PEN was measured with an ATP assay that was performed at 28 Days *In Vitro* (DIV) on rat cortical cultures grown on the material and the materials utilized in the microfabrication demonstrated excellent compatibility. The membrane bow as a result of lamination of PET-based biosensors that had to be controlled due to the post processing requirement for the creation of the microelectrodes measured lower than 15.75 µm across a large area. This measurement across several devices proved sufficient for post processing. Optimized metal deposition and definition techniques for PET-based biosensor arrays demonstrated >90% electrode yield and nano-porous platinum was electrodeposited on these sensors for low impedance performance which is necessary for network electrophysiology. Further the RMS noise of these electrodes was measured as 2–3 µV. Qualitative optical assays for the identification of polystyrene microspheres and growth of rat cortical neural cells for 7 DIV were performed successfully on the PET-based biosensor arrays.

In the future, we plan to run network electrophysiology assessments beyond proof of concept experiments for pharmaceutical compound evaluation on both PET- and PEN-based biosensors. Additionally, we plan to explore other opportunities for MEAs beyond network electrophysiology such as agricultural testing and environmental analysis. We believe such cost-effective and large area microfabrication and assembly processes are key toward achieving the goal of disposable MEAs for a wide variety of applications in neuroscience, cardiology, stem cell evaluation, toxicity testing, compound screening and other types of assays in agriculture and environmental testing.
